# Characteristics, injuries, and clinical outcomes of geriatric trauma patients in Japan: an analysis of the nationwide trauma registry database

**DOI:** 10.1038/s41598-020-76149-4

**Published:** 2020-11-05

**Authors:** Yukari Miyoshi, Yutaka Kondo, Yohei Hirano, Tadashi Ishihara, Koichiro Sueyoshi, Ken Okamoto, Hiroshi Tanaka

**Affiliations:** Department of Emergency and Critical Care Medicine, Juntendo University Urayasu Hospital, 2-1-1 Tomioka, Urayasu, Chiba 279-0021 Japan

**Keywords:** Health care, Medical research

## Abstract

Geriatric trauma is a major socio-economic problem, especially among the aging Japanese society. Geriatric people are more vulnerable to trauma than younger people; thus, their outcomes are often severe. This study evaluates the characteristics of geriatric trauma divided by age in the Japanese population. We evaluated trauma characteristics in patients (n = 131,088) aged ≥ 65 years by segregating them into 2 age-based cohorts: age 65–79 years (65–79 age group; n = 70,707) and age ≥ 80 years (≥ 80 age group; n = 60,381). Clinical characteristics such as patient background, injury mechanism, injury site and severity, treatment, and outcome were examined. Injuries among men were more frequent in the 65–79 age group (58.6%) than in the ≥ 80 age group (36.3%). Falls were the leading cause of trauma among the 65–79 age group (56.7%) and the ≥ 80 age group (78.9%). In-hospital mortality was 7.7% in the 65–79 age group and 6.6% in the ≥ 80 age group. High fall in the ≥ 80 age group showed 30.5% mortality. The overall in-hospital mortality was 11.8% (the 65–79 age group, 12.3%; the ≥ 80 age group, 11.2%). Most hospitalized patients were transferred to another hospital (the 65–79 age group, 52.5%; the ≥ 80 age group, 66.2%). We demonstrated the epidemiological characteristics of Japanese geriatric trauma patients. The overall in-hospital mortality was 11.8%, and fall injury in the ≥ 80 age group required caution of trauma care.

## Introduction

Japan, with a population of 126.167 million, has 35.885 million aged 65 and above (approximately 28.4% of the population by October 1, 2019), which is one of the largest aged populations worldwide^1^. Trauma is one of the major causes of morbidity and mortality in the elderly, making it a major socioeconomic problem. The geriatric populations are more vulnerable to trauma than the younger population. According to previous studies, mortality was significantly higher in geriatric populations than those of younger for both major and minor trauma^2,3^. Moreover, their functional activity tends to decrease after trauma, and they need long hospitalization for rehabilitation and are forced to alter their lifestyles^3,4^. However, there are only a few data on the epidemiology, clinical practice, and outcomes of geriatric trauma.

This study evaluated the characteristics and tendency of geriatric trauma in the Japanese population by dividing the geriatric population into two age-based cohorts.

## Methods

### Study design and data collection

This was a multicenter retrospective cohort study of patients treated between 2004 and 2017 using data from the Japan Trauma Data Bank (JTDB)^5^. JTDB is a nationwide trauma registry established in 2003 by the Japanese Association for the Surgery of Trauma (Trauma Registry Committee) and the Japanese Association for Acute Medicine (Committee for Clinical Care Evaluation). JTDB includes data on demographics, vital signs on arrival, cause and severity of the injury, intervention, mortality, and disposition of traumatic patients. During the study period, 264 major emergency hospitals, including approximately 95% of the tertiary emergency medical center in Japan, participated in the JTDB^6^.

The study was approved (number 29-061) by the ethics committee of the Juntendo University Urayasu Hospital. Because of the anonymous nature of the data, the requirement for informed consent was waived. We performed this study following the STROBE statement^7^.

### Study participants

We included geriatric trauma patients of all severity aged ≥ 65 years. The exclusion criterion was missing age information. Eligible patients were divided into two groups: 65–79 years (65–79 age group) and ≥ 80 years (≥ 80 age group), based on previous articles^3,8–10^.

### Variables and outcomes

For this study, we examined the following patient demographics: age, sex, vital signs on arrival, cause of injury, abbreviated injury scale (AIS) of each component, injury severity score (ISS), focused assessment with sonography for trauma (FAST) treatments, admission, mortality, and discharge status.

The AIS provides standardized terminology to describe injuries and codes the type of trauma and anatomical severity, and the severity is rated on a 6-point scale (Supplemental file 1). The AIS classifies a patient's injury site into 9 lesions: Head, Face, Neck, Thorax, Abdomen and Pelvis, Spine, Upper extremity, Lower extremity, and Unspecified. AIS codes were recorded using AIS 90 Update 98^11^. Our study included only the highest AIS score in the lesion, and multiple lesions of injuries were all included in the analysis^12^.

The primary outcome of this study was in-hospital mortality. The secondary outcomes were discharge status and mortality for each cause of injury. For subgroup analysis, we analyzed the associations of ISS with mortality between the 65–79 age and ≥ 80 age groups.

### Statistical analysis

Continuous and ordinal variables were expressed as medians and interquartile range (IQR). Categorical variables were expressed as counts and percentages. All statistical analyses were performed using IBM SPSS version 26 (IBM Corp., Armonk, NY, USA).

## Results

**Figure 1:**
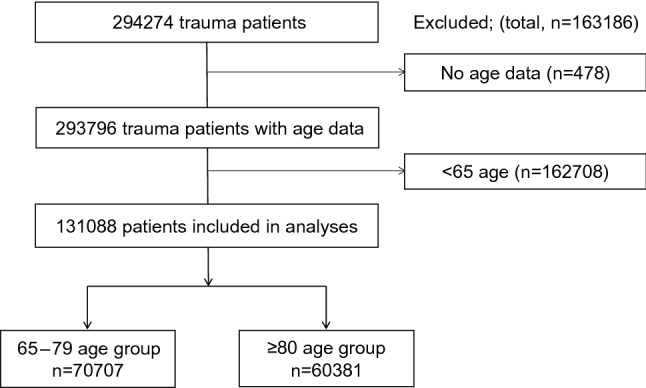
Study flow diagram of the included geriatric trauma patients.

Among the total 294,274 trauma patients during the study period, we examined 131,088 geriatric trauma patients (70,707 patients in the 65–79 age group and 60,381 patients in the ≥ 80 age group) (Fig. 1). The whole trauma patients were 24.1% (n = 70,707/293,796) of the 65–79 age group and 20.6% (n = 60,381/293,796) of the ≥ 80 age group. In total, 44.6% (n = 131,088/293,796) were geriatric in whole trauma.

Men accounted for majority of the study group but were more frequent in the 65–79 age group (58.6%) than in the ≥ 80 age group (36.3%). Fall caused most injuries in both groups (age 65–79, 56.7% and age ≥ 80, 78.9%). The proportion of head injuries of all patients was 37.0%. The median AIS score of the head region was 4, whereas other regions were in the range 1 to 3. The ISS score and FAST positive rate were relatively higher in the 65–79 age group (65–79 age group; 13 vs. ≥ 80 age group; 9 and 65–79 age group; 5.3% vs. ≥ 80 age group; 2.9%, respectively). The baseline characteristics of each group are shown in Table 1.

**Table 1:** Comparison of the baseline characteristics of the 65–79 age group and the ≥ 80 age group.

	65–79 age (n = 70,707)	≥ 80 age (n = 60,381)
Age	72 (68, 76)	85 (82, 89)
Sex, male	41,443/70,665 (58.6)	21,881/60,330 (36.3)
Systemic blood pressure on arrival	142 (119, 165)	147 (124, 168)
Diastolic blood pressure on arrival	80 (67, 92)	77 (65, 89)
Heart rate	81 (70, 94)	80 (70, 93)
Respiratory rate	20 (16, 24)	20 (16, 23)
Temperature	36.4 (35.9, 36.8)	36.6 (36.1, 37.0)
GCS	15 (13, 15)	15 (14, 15)
**Cause of injury**		
Traffic accident	22,272/67,650 (32.9)	9131/58,080 (15.7)
Fall	38,339/67,650 (56.7)	45,835/58,080 (78.9)
Sport	220/67,650 (0.33)	29/58,080 (0.05)
Compression	655/67,650 (0.97)	165/58,080 (0.28)
Other blunt trauma	2857/67,650 (4.2)	1277/58,080 (2.2)
Penetrate	1647/67,650 (2.4)	505/58,080 (0.87)
Burn	1660/67,650 (2.5)	1138/58,080 (2.0)
**AIS**		
Head (n = 48,396)	4 (3, 4)	4 (3, 4)
Face (n = 13,890)	1 (1, 2)	1 (1, 2)
Neck (n = 1166)	2 (1, 3)	2 (1, 3)
Thorax (n = 26,741)	3 (3, 4)	3 (3, 4)
Abdomen and pelvis (n = 7737)	3 (2, 3)	3 (2, 3)
Spine (n = 20,608)	3 (2, 4)	3 (2, 3)
Upper extremity (n = 22,231)	2 (2, 2)	2 (1, 2)
Lower extremity (n = 60,825)	3 (2, 3)	3 (3, 3)
Unspecified (n = 5667)	1 (1, 3)	1 (1, 3)
ISS	13 (9, 21)	9 (9, 16)
**FAST**		
Positive	3426/64,155 (5.3)	1586/54,510 (2.9)
Negative	34,099/64,155 (53.2)	16,742/54,510 (30.7)
Not conducted	26,630/64,155 (41.5)	36,182/54,510 (66.4)

GCS, Glasgow coma scale; AIS, abbreviated injury scale; ISS, injury severity score; FAST, focused assessment with sonography for trauma.

Missing data: Sex = 93, Cause of Injury = 5358, FAST = 12,423. Data are presented as median (interquartile range) except for gender, cause of injury, and FAST, which is presented as numbers (%).

As for treatment, bone fixation was performed at a higher number in the ≥ 80 age group than the 65–79 age group. The overall in-hospital mortality was 11.8%, and the emergency department mortality was 3.2%. In-hospital mortality was slightly higher in the 65–79 age group than in the ≥ 80 age group (12.3% and 11.2%, respectively). Of the total patients, only 37.4% of the patients were discharged ( 65–79 age group, 45.5% and ≥ 80 age group, 28.2%), and the others were transferred to another hospital or facility (65–79 age group, 52.5% and ≥ 80 age group, 66.2%). The comparison of interventions and outcomes of each group are shown in Table 2.

**Table 2:** Comparison of interventions and outcomes of the 65–79 age and ≥ 80 age group.

	65–79 age (n = 70,707)	≥ 80 age (n = 60,381)
**Treatments**		
Craniotomy	2792	1231
Craterization	1225	1026
Thoracotomy	965	430
Celiotomy	1657	559
Bone fixzation	17,110	24,074
TAE	1943	1281
Blood transfusion	10,016/67,050 (14.9)	6977/57,496 (12.1)
**Admission**		
ICU	38,462/65,490 (58.7)	22,449/56,391 (39.8)
General word	24,500/65,490 (37.4)	32,536/56,391 (57.7)
Died at ED	2528/65,490 (3.86)	1406/56,391 (2.5)
In-hospital mortality	8055/65,278 (12.3)	6345/56,428 (11.2)
**Discharged place**		
Home	26,048/57,223 (45.5)	14,133/50,083 (28.2)
Another hospital	30,068/57,223 (52.5)	33,179/50,083 (66.2)
Others	1107/57,223 (1.9)	2771/50,083 (5.5)

TAE, transcatheter arterial embolization; ICU, intensive care unit; ED, emergency department.

Missing: Blood transfusion = 6542, Admission = 9207, In-hospital mortality and Discharged place = 9382. Data are presented as numbers (%).

Table 3 shows the mortalities of the 65–79 age group and ≥ 80 age group with the cause of injury. For the 65–79 age group and ≥ 80 age group, traffic accidents and burns were the cause of injury with high mortalities, especially in the ≥ 80 age group (16.3% vs. 24.7% and 23.3% vs. 33.9%, respectively). In contrast, falls as well as traffic accidents contributed mostly to trauma deaths in the 65–79 age group, although its mortality was relatively low.

**Table 3:** Mortality rates of the 65–79 age group and ≥ 80 age group with the cause of injury.

	65–79 age (n = 70,707)	≥ 80 age (n = 60,381)
**All cause (n = 125,730)**	7626/67,650 (11.3)	5990/58,080 (10.3)
Traffic accident (n = 31,403)	3620/22,272 (16.3)	2255/9131 (24.7)
Fall (n = 84,174)	2963/38,339 (7.7)	3035/45,835 (6.6)
Sport (n = 249)	1/220 (0.5)	0/29 (0)
Compression (n = 820)	133/655 (20.3)	45/165 (27.3)
Other blunt trauma (n = 4134)	361/2857 (12.6)	199/1277 (15.6)
Penetrate (n = 2152)	162/1647 (9.8)	70/505 (13.9)
Burn (n = 2798)	386/1660 (23.3)	386/1138 (33.9)

Missing: All cause = 5358. Data are presented as numbers (%).

Table 4 shows the mortalities of the 65–79 age group and ≥ 80 age group with subclassification of fall injury. High fall was the highest mortality in the groups, although less common than stair-fall and ground-level fall.

**Table 4:** Mortalities of the 65–79 age and ≥ 80 age group with subclassification of fall injury.

	65–79 age (n = 38,339)	≥ 80 age (n = 45,835)
**Total fall injuries (n = 84,174)**		
High fall (n = 6913)	1138/5367 (21.2)	472/1546 (30.5)
Stair-fall (n = 17,403)	891/11,150 (8.0)	711/6253 (11.4)
Ground level fall (n = 59,858)	934/21,822 (4.3)	1852/38,036 (4.9)

Data are presented as numbers (%).

**Figure 2:**
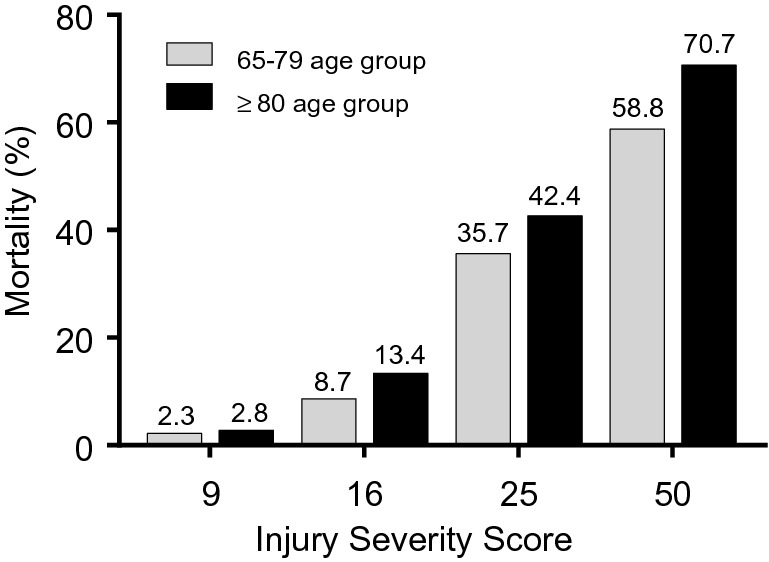
Relationship between mortality and injury severity score in the 65–79 and ≥ 80 age groups.

Figure 2 shows increased mortality in the ≥ 80 age group in ISS of 9, 16, 25, and 50, compared to the 65–79 age group.

## Discussion

This study showed the epidemiological characteristics of geriatric trauma patients in Japan. The overall in-hospital mortality was 11.8%, and most hospitalized patients were transferred to another hospital. The leading cause of geriatric trauma was fall, which was seen in 56.7% for the 65–79 age group and 78.9% for the ≥ 80 age group. Regarding in-hospital mortality, fall injuries were 7.7% mortality in the 65–79 age group and 6.6% mortality in the ≥ 80 age group. High fall in the ≥ 80 age group was the highest mortality, with 30.5%.

Consistent with previous studies, men tended to experience trauma more than women in the 65–79 age group, whereas women were more commonly affected in the ≥ 80 age group^8,9^.

Falls were the leading cause of trauma in the 65–79 age group. Previous studies have shown that falls accounted for nearly three-quarters of all the traumas in the geriatric population, with motor vehicle accidents accounting for nearly all the remaining traumas^10,13^. We found more traffic injuries in the 65–79 age group and more falls in the ≥ 80 age group.

This study also showed that head injuries were the most frequent and severe cause. More importantly, falls are the leading cause of traumatic brain injury among older adults^14^. A previous study mentioned a linear relationship between age and mortality after head trauma^15^. Therefore, the 65–79 age population needs to adopt more precautions for fall injury prevention and treatment than the younger population. Although burns are less frequent, the mortality rate is high, and therefore, caution is necessary for prevention.

In-hospital mortality of geriatric trauma patients varied from 9.9 to 26.8% partly because of differences in the definition of geriatric and inclusion criteria such as the severity of trauma^10,16,17^. In this study, the overall in-hospital mortality rate was 11.8%, and the emergency department mortality rate was 3.2%. Sammy et al.^18^ indicated that a significant secondary increase in the risk-adjusted mortality was observed among older adults, whereas our study showed no remarkable difference in mortality between the two age groups. This difference may be because our study included all severities and mechanisms of injury. Thus, we performed subgroup analysis and compared the mortality in each ISS value. The ≥ 80 age group has increased mortality in every ISS value. Frailty is a clinical condition characterized by a decrease of homeostatic reserves and is responsible for enhanced vulnerability to endogenous and/or exogenous stressors^19^. Our study demonstrated that frailty affected in-hospital mortality of geriatric trauma at each ISS of 9, 16, 25, and 50. Frailty increased in-hospital mortality even in not severe trauma.

Another issue of concern besides death due to geriatric trauma is that although most patients survive, they often need careful and long-term treatment; patients are transferred and forced to alter their lifestyles. Only 28.2% of the ≥ 80 age group could be discharged at home in our results. Further investigation is needed about the information on rehabilitation, functional status of at or after discharge, and quality of post-trauma life. Prevention of fall injuries has considerable potential for reducing morbidity, mortality, and medical costs. This study showed that “geriatric trauma” has different characteristics depending on age. Therefore, epidemiology-based studies for the prevention and treatment of trauma among the geriatric should be continued.

There are some strengths to our study. First, we used the AIS scale, a widely and internationally accepted scoring system for assessing the severity of trauma. Furthermore, the AIS score was scored by trained physicians or technicians. Second, this is a nationwide Japanese registry study. We could include and analyze many geriatrics patients and various types of trauma. Describing all types of geriatric trauma by using the AIS score in Japan is novel.

There are several limitations to this study. First, the patients were registered at each facility, and not all geriatric patients were necessarily registered in the JTDB^12^. Many patients with minor injuries might not have been included. Second, there were some important missing variables; lack of information on the cause of injury and outcomes could affect the results. Furthermore, because of missing data, classification was varied in tables. Third, the incidence rate of geriatric trauma is unknown because we could not get non-traumatic geriatric population data. A large population-based study may be needed. Finally, we could not use pre-existing disease information because of missing data. Many geriatric patients have pre-existing conditions, which may affect their mortality.

## Conclusions

We demonstrated the epidemiological characteristics of geriatric trauma patients in Japan. The overall in-hospital mortality was 11.8%, and the leading cause of trauma was fall. In-hospital mortality was higher in the ≥ 80 age group than those of 65–79 aged. Fall injury in the ≥ 80 aged patients required caution of trauma care.

## Supplementary Information

Supplementary Information.

## Data Availability

The data are available from the corresponding author upon reasonable request.
