# Supplementary material for: Characteristics, injuries, and clinical outcomes of geriatric trauma patients in Japan: an analysis of the nationwide trauma registry database
Source: Sci Rep. 2020 Nov 5;10:19148. doi: 10.1038/s41598-020-76149-4 (PMC7645585; doi:10.1038/s41598-020-76149-4)
Supplement: Supplementary file 1 — Supplementary Information. [file 41598_2020_76149_MOESM1_ESM.docx]

**Supplemental Data**

**Characteristics, Injuries, and Clinical Outcomes of Geriatric Trauma Patients in Japan: An Analysis of the Nationwide Trauma Registry Database**

Yukari Miyoshi^1^, Yutaka Kondo^1^, Yohei Hirano^1^,

Tadashi Ishihara^1^, Koichiro Sueyoshi^1^, Ken Okamoto^1^, and Hiroshi Tanaka^1^

^1^Department of Emergency and Critical Care Medicine,

Juntendo University Urayasu Hospital

Supplemental file 1. Trauma severity classification by AIS 2005, update 2008 version

| AIS point | Severity | | Description |
| --- | --- | --- | --- |
| 0 | | None | No injury |
| 1 | | Minor | Superficial injury |
| 2 | | Moderate | Reversible injury; medical attention required |
| 3 | | Serious | Reversible injury; hospitalization required |
| 4 | | Severe | Life threatening injury |
| 5 | | Critical | Non-reversible injury |
| 6 | | Maximal | Fatal injury |

AIS; abbreviated injury scale.
